# On the Danger of Inoculating with Purulent Fluids

**Published:** 1800-12

**Authors:** William H. Wollaston, John Pearson, John Griffiths, Richard Croft

**Affiliations:** London; London; London; London


### *To the Editors of the Medical and Phyfical Journal.*

Gentlemen,

Having been informed that in an inoculation of the poor of a parifh in the neighbourhood of town for the Vaccine Difeafe, fymptoms had occurred very much unlike thofe we have ever feen or heard of in the Cow-pox, and that one of them had actually died under the difeafe, we have been induced to vifit them; and we now feel it incumbent on us, as a duty to the public, and in juftice to Dr. Jenner, to make the following ftatement, as the refult of our enquiries.

The poor had been divided into four diftricts, to be inoculated by four different medical gentlemen refident in that parifh. The patients of three of thefe gentlemen had the Cow-pox in the regular manner, without one unfavorable fymptom.

In the remaining diftrict it was obferved, that of fifteen perfons inoculated on the 22d of October, only fix took the infection.

There is every reafon to think that this firft inoculation caufed no appearances in any degree deviating from the ufual progrefs of the Cow-pox.

The remaining nine were inoculated again on the 31ft of October.

The gentleman who performed this inoculation candidly acknowledges, that the matter with which he had charged the lancet for this purpofe had been taken at a very late period of the difeafe, after a brown fcab had formed, and had a purulent appearance at the time it was taken from the arm, confequently had, by degenerating, loft the power of giving the Cow-pox infection, and appears to have been the caufe of all the mifchief that enfued.

It is proper however to remark, that the matter thus taken was not alone trufted to in the dry ftate, but that, in order to enfure enfure fuccefs, frefh Cow-pox *fluid* was alfo taken upon the fame lancet from the arms of different children before inoculated.

The fymptoms produced by this inoculation were extenfive eryfipelas, fpreading rapidly from the part inoculated, accompanied in many inftances by confiderable conftitutional affection, followed in moft by an immediate ulcerative procefs, and in fome even a tendency to gangrene.

In confirmation of the opinion, that an infection was thus propagated, diftinct from that of the Cow-pox, we are alfo under the neceffity of ftating, that in two of the patients who are believed to have had the Cow-pox diftinctly by the former inoculation, the fame fymptoms were unfortunately induced, in attempting to procure Cow-pox *fluid* from them with the fame lancet.

We have, however, the fatisfaction of adding, though feveral remain indifpofed, that all are in a fair way of recovery.

WILLIAM H. WOLLASTON, M. D.

JOHN PEARSON,

JOHN GRIFFITHS,

*London, Nov.* 20, 1800. RICHARD CROFT.

P. S. For the information of the public, and as a caution to thofe who may need to be reminded of the injunctions already given by Dr. Jenner, it is thought advifable to repeat, as concifely as may be, the directions which may be collected from his various publications on this fubject.

1. The Cow-pox *fluid* fhould be taken not later than the ninth day of the difeafe.

2. The *fluid* fhould be perfectly tranfparent, as it is not to be depended upon if it has become in any degree opaque.

3. The *fluid*, if not ufed immediately, fhould be allowed to dry gradually and thoroughly before it is laid by for future ufe.

4. The punctures can fcarcely be made too fuperficial, and on no account fhould more than one be made in each arm.

5. Attention fhould be paid to reprefs, as foon as may be, any excefs of inflammation that may happen to arife.

*** During a converfation with a profeffional gentleman of the firft rank and eminence in the metropolis, on the fubject of the decompofition of the Vaccine fluid, I was informed, that many years ago he inoculated a child with Variolous matter fo very far advanced, that he took it from under a fcab. It produced a very violent eryfipelatous inflammation on the arm, which gradually extended almoft over the whole body. The general indifpofition thus excited was fevere, the arm ulcerated, and the difeafe terminated in an anafarcous fwelling of the left leg and thigh, and lafted fix months. It yielded at length to fea-bathing, when the child was again inoculated with

*perfect*

*perfect* variolous matter, which produced the ſmall-pox as completely as if the conſtitution had not felt the influence of the imperfect.

Is the general maſs of the human fluids, or thoſe of quadrupeds, capable of undergoing changes ſpontaneouſly, which can produce theſe effects? Or, do they depend upon their degeneracy from matter once poſſeſſing ſpecific properties? Wounds of the ſkin inflicted during diſſection, I believe, ſometimes produce effects not very diſſimilar from thoſe above recited. EDIT.

[ *See Dr. Lettsom's Leter on the same subject, p.* 567. ]

